# Cellulose Cryogels as Promising Materials for Biomedical Applications

**DOI:** 10.3390/ijms23042037

**Published:** 2022-02-12

**Authors:** Irina V. Tyshkunova, Daria N. Poshina, Yury A. Skorik

**Affiliations:** Institute of Macromolecular Compounds of the Russian Academy of Sciences, Bolshoi VO 31, 199004 St. Petersburg, Russia; tisha19901991@yandex.ru (I.V.T.); poschin@yandex.ru (D.N.P.)

**Keywords:** cellulose, cellulose cryogel, freeze-drying, tissue engineering, regenerative medicine

## Abstract

The availability, biocompatibility, non-toxicity, and ease of chemical modification make cellulose a promising natural polymer for the production of biomedical materials. Cryogelation is a relatively new and straightforward technique for producing porous light and super-macroporous cellulose materials. The production stages include dissolution of cellulose in an appropriate solvent, regeneration (coagulation) from the solution, removal of the excessive solvent, and then freezing. Subsequent freeze-drying preserves the micro- and nanostructures of the material formed during the regeneration and freezing steps. Various factors can affect the structure and properties of cellulose cryogels, including the cellulose origin, the dissolution parameters, the solvent type, and the temperature and rate of freezing, as well as the inclusion of different fillers. Adjustment of these parameters can change the morphology and properties of cellulose cryogels to impart the desired characteristics. This review discusses the structure of cellulose and its properties as a biomaterial, the strategies for cellulose dissolution, and the factors affecting the structure and properties of the formed cryogels. We focus on the advantages of the freeze-drying process, highlighting recent studies on the production and application of cellulose cryogels in biomedicine and the main cryogel quality characteristics. Finally, conclusions and prospects are presented regarding the application of cellulose cryogels in wound healing, in the regeneration of various tissues (e.g., damaged cartilage, bone tissue, and nerves), and in controlled-release drug delivery.

## 1. Introduction

**Figure 1:**
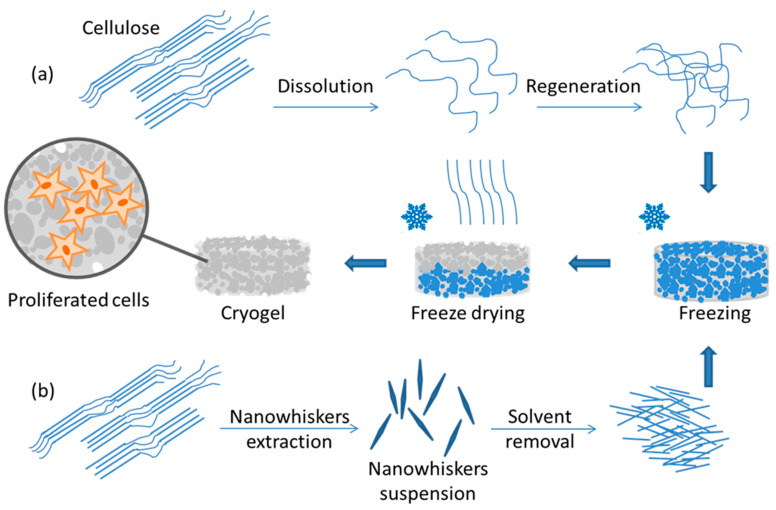
Ways to produce cellulose cryogel for biomedical applications: (**a**) via dissolution and regeneration; (**b**) using nanowhiskers.

Cryogelation is one of the newly developed protocols for the production of polysaccharide materials for biomedical purposes [1]. Polysaccharide-based cryogels form a spongy super-macroporous structure during freeze-drying, making them highly promising materials for tissue engineering [2]. The production of polysaccharide cryogels has recently become a popular approach for the development of scaffolds [3], and these matrices are readily obtained by dissolving a polysaccharide (usually cellulose) in an appropriate solvent, followed by polymer regeneration from solution (solvent removal), freezing, and freeze-drying. Figure 1 shows the scheme for producing cellulose cryogels.

At the regeneration step, the polymer passes from the dissolved state to an insoluble state, and subsequent freezing leads to ice crystal formation. The removal of the ice during freeze-drying then generates pores and leaves a cryogel with a complex three-dimensional structure [4]. The high porosity and hydrophilicity, high water retention capacity, interconnectedness of the pores, and material consistency make cryogels very similar to natural soft tissues [5], while their mechanical stability allows their use in vivo [6]. Cryogels can also stimulate the in vivo production of various natural molecules, including antibodies, and they can act as in vitro bioreactors for the expansion of cell lines and as a means of cell separation. Excellent in vivo results have been obtained using cryogels as scaffolds for tissue engineering, as cryogels can promote the resumption of growth in numerous damaged tissues [3]. However, the surface properties of tissue engineering materials affect cell affinity [1], and these properties depend on a large number of different factors, including the conditions used for polysaccharide dissolution, regeneration, and freezing.

Biocompatibility is one of the main requirements for cryogels used as scaffolds. The ideal scaffold should be porous, biodegradable, biocompatible, and bioresorbable and should not trigger an immune response or inflammation [7]. Consequently, scaffolds made from natural polymers have several advantages over those made from synthetic polymers, as natural polymers are bioresorbable and biocompatible, have low immunogenicity and cytotoxicity, and can stimulate intercellular interactions. By contrast, the degradation of synthetic polymers can generate harmful by-products and can have problems in terms of injection and infection [1]. Natural biopolymers, particularly cellulose, have therefore become very popular materials for the preparation of porous products used for biomedical purposes, such as wound healing, tissue engineering, and drug delivery [8,9].

Cellulose has found particular favor in biomedical sciences due to its mechanical strength, biocompatibility, and hydrophilicity, making it a promising polysaccharide for the production of biocompatible porous cryogels [10,11,12]. Cellulose-based materials have been proposed for a variety of biomedical applications [13,14] because, unlike other polysaccharides, cellulose is relatively bioinert and is not biodegraded in the human body. Thus, newly regenerated tissue cannot displace a cellulosic scaffold, which can be an advantage in tissue engineering. Cellulose materials have found applications in the regeneration of bone [15], neural [16], and cartilage [17] tissues, as well as in wound dressings [18]. The bioinertness of cellulose also meets the requirement that a scaffold material should not induce foreign body responses [19].

This review considers the preparation of cellulose-based cryogels using the freeze-drying technique, and presents data on the use of these cryogels for biomedical purposes. The structural features and properties of cellulose and the difficulties associated with the dissolution of cellulose are reviewed. Information on the methods for dissolving cellulose and producing cellulose cryogels is presented. The influence of various factors on the structure and properties of the produced materials is discussed, and the advantages of the freeze-drying process are analyzed. Recent studies on the production and use of cellulose cryogels for biomedical purposes are summarized, and the main quality parameters of these cryogels are presented. The current status and prospects for the use of cryogels in tissue engineering are discussed.

Previously published reviews on the biomedical application of cryogels have provided much information on various polysaccharide cryogels, but cellulose cryogels have received relatively little attention. The available reviews on cellulose cryogels contain information on production methods and the characterization of properties and morphology, without indicating possible directions for biomedical application [20,21]. Other reviews consider cellulose cryogels to be sorbents [12,22]. Reviews that focus on the biomedical applications of cryogels contain information on many polysaccharide cryogels, with little [2,3,23,24] or no mention of cellulose cryogels [1]. A review of nanocellulose sponges and their biomedical applications has been published [25]. By contrast, we present data on the use of different cellulose types (cellulose of various origins). This review is especially focused on analyzing data on cellulose cryogels obtained by freeze-drying, offering information on the use of various cellulose types for producing biomedical cryogels. The information presented starts with the structural features of cellulose and its solvents for the production of cryogels and ends with data on the biomedical applications of various cellulose cryogels.

## 2. Cellulose as a Source for Producing Biomedical Materials

Cellulose is a promising raw material for the production of functional biomedical materials [10]. Cellulose can be shaped in many different ways: into beads [26], fibers with a diameter from tens of nm to tens of microns [27], films (cellophane), porous foams (sponges), and aerogels [20,28,29]. The morphology and properties of these objects can be very different.

Cellulose, as a biomedical material, has certain advantages over other traditional biopolymers, including its prevalence (it can be isolated from various natural materials), availability, low toxicity, renewability, and biocompatibility, making the development of cellulose-based cryogels a promising research direction [30]. The freeze-drying of cellulose hydrogels imparts a complex heterogeneous structure to cellulose, creating useful building blocks for complex hierarchical structures [31]. Porous cellulose materials are very attractive for a variety of biomedical applications, including controlled drug release, tissue engineering scaffolds, matrices for cell growth, biosensors, and antibacterial wound dressings [12,31,32,33,34,35]. Each of these applications requires materials with a specific morphology, pore size distribution, specific surface area, and material density. However, the complex supramolecular structure of cellulose creates difficulties in its dissolution and processing into biomedical products.

**Figure 2:**
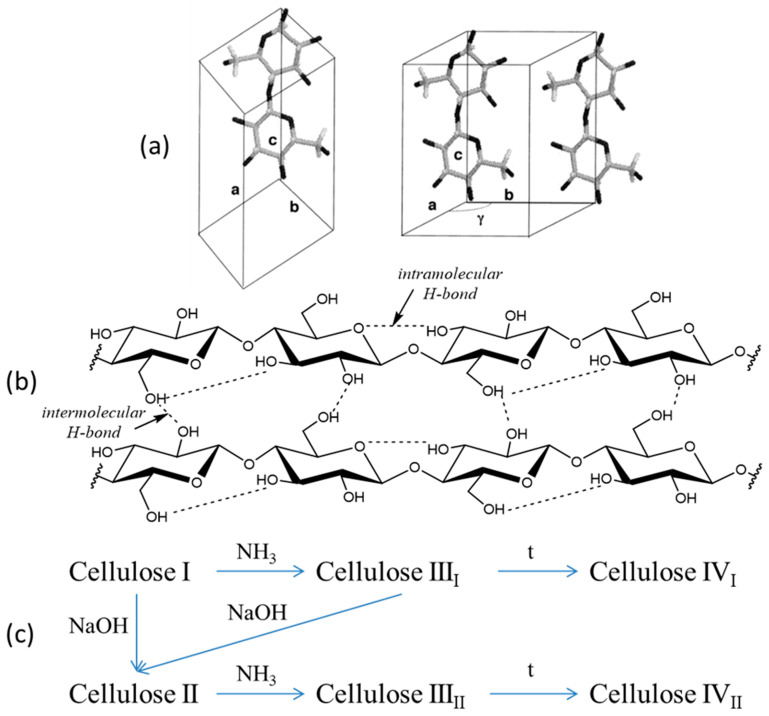
Complex molecular structure of cellulose: (**a**) native cellulose I unit cells, triclinic Iα and monoclinic Iβ [37] © 2022 National Academy of Sciences; (**b**) H-bond network of cellulose I; (**c**) polymorph transitions.

Cellulose consists of anhydroglucose units (C_6_H_10_O_5_) linked by β-glycosidic (1 → 4) bonds and has a high crystalline content [36]. The hydroxyl groups in the cellulose macromolecule are involved in intra- and intermolecular hydrogen bonds (Figure 2b), which lead to the formation of various ordered crystal structures.

Crystalline allomorphs of cellulose I, II, III, and IV are distinguished according to their X-ray diffractometry and solid-state ^13^C NMR spectra. Cellulose I, the most abundant form in nature, is a crystalline native cellulose, whereas cellulose II is obtained by mercerization (alkaline treatment) or regeneration (solubilization and subsequent recrystallization) (Figure 2c). Celluloses III_I_ and III_II_ can be formed from celluloses I and II, respectively, by treatment with liquid ammonia, and the reaction is reversible [38]. Cellulose IV_I_ and IV_II_ can be obtained by heating celluloses III_I_ and III_II_, respectively [39]. Lightweight porous materials can be obtained from celluloses I or II [12], but most research has focused on cellulose I. The crystalline structure of cellulose I is a mixture of two different crystalline forms: cellulose Iα (triclinic) and Iβ (monoclinic) (Figure 2a) [40]. The relative amounts of cellulose Iα and Iβ vary depending on the cellulose source (for example, the Iβ form is dominant in higher plants, whereas the Iα form is typically found in algae and bacteria). Cellulose crystallites are usually about 5 nm wide; however, these crystallites are imperfect, and part of the cellulose structure is less ordered, termed amorphous. The traditional two-phase cellulose model describes cellulose chains containing both crystalline (ordered) and amorphous (less ordered) regions [41]. 

This added complexity of the supramolecular structure of cellulose creates difficulties in its dissolution and processing. Typical cellulose solvents include 7–9% aqueous NaOH [26,42,43], Cu-ethylenediamine (or Cd-ethylenediamine) complexes [44], LiCl/dimethylacetamide (DMAc) [45], *N*-methyl-morpholine-*N*-oxide monohydrate [46,47,48,49], molten salt hydrates, and ionic liquids [50,51,52,53] (Table 1).

However, most of these are toxic, have only limited ability to dissolve high molecular weight cellulose, and are difficult to remove from the final product [55]. The processing steps required for dissolution, gelation, and solvent removal for cellulose cryogel formation are very slow and can take several days [56]. However, one advantage of the insolubility of cellulose in water and typical organic liquids is that, with proper reinforcement, the structure of lightweight cellulose materials can be retained when they are immersed in most liquids [57].

Interest in porous biomedical materials continues to grow, as evidenced by the number of publications each year [2,58,59,60]. Cellulose is a promising raw material for the production of cryogels.

## 3. Advantages of Freeze-Drying and Factors Affecting the Structure and Properties of Cryogels 

Freeze-drying allows the preservation of the micro- and nanostructure of the material and the generation of a large specific surface area (up to 300 m^2^/g) in the dried state [14,61]. One advantage of freeze-drying is that it has no requirement for the use of flammable liquids (e.g., ethanol/acetone that are required for supercritical drying, which also allows preservation of the nanostructure of the material); another is that the structure of the resulting material corresponds to the structure of the frozen dispersion [62]. Ice crystals formed during the freezing of the dispersion change the distribution of particles, and subsequent drying creates pores where the ice crystals had formed [63]. The morphology of the materials obtained by freeze-drying can vary from random networks to lamellar solid structures. These different types of network structures can produce equally lightweight materials; therefore, they can be easily designed and produced with environmental friendliness and safety in mind.

**Figure 3:**
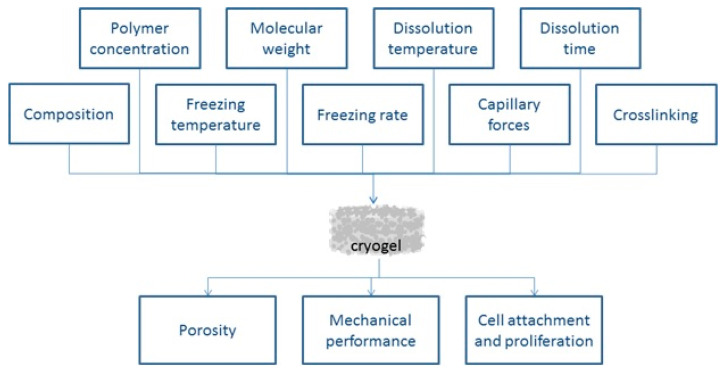
Influence of different factors on cellulose cryogel properties.

The final properties of a cryogel, including its biocompatibility, mechanical, and thermal properties, and degradability, depend on many factors (Figure 3). 

The chemical composition of the cryogel is probably the most important factor, since it determines the biocompatibility and degradability of the cryogel and, to some extent, affects the mechanical and thermal properties of the cryogel. The porosity and degree of crosslinking mainly affect the mechanical properties, while crosslinking itself affects the biocompatibility and degradability of the cryogel [2]. The pore size, wall thickness, and density affect the properties of cryogels [64], as thicker and higher-density walls improve their mechanical properties. The thickness and density, in turn, depend on the concentration of the polymer and the type of crosslinking in the cryogel.

The production processes used to form the cryogels also affect their structure. For example, an increase in the freezing rate or a decrease in the cryogelation temperature decreases the cryogel pore size because the solvent freezes at a higher rate, allowing for the growth of only a small number of ice crystals [65,66]. Further, a temperature gradient occurs during cryogelation, which leads to a non-uniform pore size distribution [67]. Initially, the external part of the sample is exposed to a low temperature, which leads to an increase in the freezing rate and a smaller pore size than that subsequently formed in the internal cryogel material. However, this heterogeneous pore size distribution is not an obstacle to the use of cryogels in tissue engineering, since many tissues of the human body also have heterogeneous morphology [68]. Cryostructuring, including directional freezing of cryogels, has been used to achieve varying degrees of porosity (45–75%, pore size 70–85 nm) or to equalize the porosity or anisotropy within cryogels [60].

The mechanical properties of cryogels are commonly evaluated using compression testing [3]. Reducing the pore size of cryogels has been reported to increase compressive strength [69], whereas increasing porosity increases the compressive deformation of the cryogel [70]. For one type of cryogel (injection cryogels), low compression deformation is undesirable; their ideal porosity is 91% [67].

Cryogels used in biomedical applications may require that the material eventually degrade within the body, but cryogels still need to perform their functions before this degradation occurs. Therefore, knowledge of the changes in the mechanical properties of cryogels throughout their degradation would be useful [71]. The thickness of the cryogel walls is assumed to decrease during enzymatic degradation, and in some cases, the walls are destroyed. Whether this process occurs for cryogels degraded by other mechanisms (e.g., by cleavage of disulfides [72] or chemical hydrolysis [59]) is unclear. The degradation of cryogels leads to an increase in pore size, possibly due to thinning of the pore walls and a decrease in crosslinking [73]. The mechanical properties of degraded cryogels are largely overlooked in the current literature, despite their importance for applications such as scaffold materials [71]. Due to their non-biodegradability in the human body, the main application areas of cellulose materials are bone tissue regeneration (bone implants) [15,66,67] and the production of wound dressings [18]. 

The following sections provide a more detailed description of some of the variables that affect the structure and properties of cryogels: the type and degree of crosslinking, the concentration and molecular weight of the polymer, the parameters of gelation and cryoconcentration, and the effects of capillary forces, temperature, and freezing rate.

### 3.1. Type and Degree of Crosslinking 

Crosslinking can provide better mechanical performance and integrity for cellulose cryogels. The type of crosslinking affects the rigidity and degree of swelling, which in turn affects the elastic and mechanical properties and pore size of the cryogel. Methods for cryogel formation include chemical crosslinking and physical gel formation using natural or synthetic polymers [65]. Chemical crosslinking occurs during the storage of the polymer solution at a given temperature, whereas physical crosslinking occurs during the thawing step, where faster thawing results in weaker gels [6]. Physical crosslinking generates cryogels with pore sizes of less than 10 µm [74,75,76,77], whereas chemical crosslinking allows for cryogels with large pore sizes of 80–200 µm [28,68,72]. One hypothesis to explain the difference in structure formation during physical and chemical gelation of cellulose is that, during physical gelation, the chains self-associate to form a heterogeneous network with “thick” walls and pores of different sizes. By contrast, during chemical gelation, chemical bonds act as separators between the chains, thereby breaking their self-association and preventing packaging. The result is a more uniform chemical network with higher swelling and transparency when wet and lower density when dry [33].

The degree of crosslinking (i.e., the ratio of monomer to crosslinking agent) in a chemically crosslinked cryogel affects its mechanical properties. Chemical crosslinking can provide good mechanical properties; however, the compounds used as crosslinkers are often toxic, difficult to remove, and not biocompatible [78]. The effect of the amount of the crosslinking agent on the mechanical properties of cellulose cryogels is debatable, as some data show an increase in the compressive modulus with an increase in the crosslinking agent concentration [79], whereas other studies indicate an increase in the storage modulus for cellulose cryogels from 45 to 675 Pa with a decrease in the crosslinking agent concentration [80]. An increase in the crosslinking agent concentration (epichlorohydrin) also results in the formation of an inhomogeneous structure of the cellulose cryogel, whereas dense areas are observed when the pore size is 200 μm [33].

The degree of crosslinking in physically crosslinked cryogels is controlled by changing the number of freeze-thaw cycles [2]. Physical crosslinking does not use any organic solvents or toxic crosslinking agents, thereby eliminating any danger of residues in the final material and making this method very promising for biomedical applications [78]. Physical crosslinking is also easier, and this translates into cost savings. The problem with physical methods is obtaining satisfactory properties without any chemical modification while maintaining biocompatibility, biodegradability, and bioactivity [78]. However, according to some data, compared to their chemically crosslinked counterparts, physically crosslinked cryogels demonstrate greater mechanical strength [81] and crystallinity (cellulose cryogels) [33].

### 3.2. Concentration and Molecular Weight of the Polymer

A minimum (critical) concentration of cellulose is required to retain the integrity (shape) of the produced cryogel (i.e., to retain the integrity of the network after removing the liquid phase) [22,77,78]. This critical concentration is probably related to percolation within the precursor network [82,83], where overlap or interaction between cellulose chains results in the formation of an autonomous network [34]. At a concentration below the critical value, the network is unstable, and shrinkage increases with decreasing cellulose concentration [83]. A cellulose concentration of more than 3% in solution is required to obtain cryogels, as studies have shown that cryogels do not form at concentrations of less than 3% [28,54]. The critical concentration of the polymer also affects the mechanical properties of the produced cryogels [84]. 

Solutions with high polymer concentrations produce cryogels with small average pore sizes. This is due to an increase in the availability of crosslinked groups and a decrease in the availability of free water. As with conventional hydrogel formation, increasing the polymer content increases the rigidity of the cryogels. An increase in the polymer concentration also leads to a decrease in porosity and swelling of the cryogel [54,85], while decreasing the degradation rate [85]. 

The molecular weight of the polymer affects the structure of the cryogel. The use of polymer solutions with a lower molecular weight at the same mass concentration in a gel solution leads to the formation of larger pores compared to the use of gel solutions of polymers with a higher molecular weight [24,54,83]. Higher molecular weight polymer solutions will generate smaller pores due to the relatively smaller volume of free water that can form ice crystals in the solution. Similar observations were recorded when producing cryogels based on cellulose with various degrees of polymerization [21] compared to the concentrations of other polymers (gelatin) in a gel solution [86,87]. 

An increase in the degree of cellulose polymerization leads to an increase in the undissolved fraction in the solution, which reduces the content of dissolved cellulose in the matrix solution. This leads to the formation of voids in the dry matter [20]. Thus, the incomplete dissolution of cellulose with a high degree of polymerization and an increase in material heterogeneity will worsen the mechanical properties of the final cellulose composites.

### 3.3. Gelation and Cryoconcentration Parameters

The temperature and dissolution time (gelation) of the polymer affect the cryogel structure and properties. These parameters are typically set to values that provide the best structure and properties for each cryogel. For example, the optimal dissolution time is 24 h at room temperature for microcrystalline cellulose [54] and 16 h at room temperature for chitin [88]. An optimum temperature also exists for gelation and cryogelation for maximization of the pore size [89]. The effect of the gelation and crystallization rate of the solution on the physical properties of cryogels therefore becomes important.

To obtain a macroporous structure of cryogel by cryogelation, the gel solution must first partially crystallize to form solidified solvent crystals (pore-forming agents). This can be complicated by the action of hydrogel components that lower the freezing point of the solution (the “freezing point lowering effect”) and by the effects of supercooling. To obtain a homogeneous macroporous hydrogel, the crosslinking rate of the polymer must be lower than the crystallization rate of the solvent [90]. If crosslinking occurs faster than the solvent can crystallize, a non-macroporous gel will form. Conversely, larger pores can be formed by reducing the crosslinking rate (the formation and growth of crystal pore-forming agents). The inhibitory effect of supercooling during solvent crystallization can be overcome by increasing the cooling rate. This increase leads to the formation of smaller [91] or even irregular pores [92], depending on the extent of the increase in the cooling rate.

The cryoconcentration of components in the liquid phase also affects the process of cryogel formation. For example, cryoconcentration lowers the critical concentration required for gelation, thereby allowing gel solutions with low monomer content that would not normally set at room temperature to set under cryo-conditions. Cryoconcentration can also speed up the gelation process [6]. The effect of cryoconcentration on the mechanical properties of the cryogel is of interest, given that the compaction of the polymer in the pore walls significantly increases local mechanical properties, such as elasticity.

### 3.4. Capillary Forces

The capillary forces between the particles of a porous material affect its density. A decrease in capillary forces decreases the density of the material, resulting in lighter materials [93]. Freeze-drying avoids capillary forces; for example, freezing at −18 °C and subsequent freeze-drying produced the lightest cellulose material (density 0.0002 g/cm^3^) from a 0.1% cellulose nanofibril hydrogel [94]. The cooling rate is lower for this type of freezing (−18 °C) than when liquid nitrogen is used for freezing. This promotes the growth of ice crystals and produces a material of lower density [83,94].

### 3.5. Freezing Parameters

The freezing temperature affects the cryogel morphology and can result in the formation of a lamellar structure and highly porous gels with preserved micro- and nanostructure [61]. Smaller pores can be formed by lowering the temperature [95]. At lower temperatures, the solvent crystallizes more rapidly, resulting in the formation of smaller solvent crystals (pore-forming agents). However, due to the increased crystallization of the solvent, the liquid microphase becomes more concentrated, which leads to the formation of thinner and denser pore walls. A 15 °C decrease in the freezing point has been shown to cause a decrease in the pore diameter of polyacrylamide cryogels by an average of 30 μm [96]. By contrast, the pore sizes of cryogels based on polyvinyl alcohol, laminin, or gelatin crosslinked with glutaraldehyde were unaffected by the freezing point [65]. Freezing at −20 °C resulted in the formation of lamellar structures with few pores. A decrease in the pre-freezing temperature to −80 °C and −196 °C led to the appearance of more porous structures. In general, a lower pre-freezing temperature produces a more porous and less agglomerated cryogel structure [95]. 

Rapid cooling of the dispersion is effective for producing numerous and small ice crystals and leads to the formation of small pores (hence, a high specific surface area) [83,97]. The effects of temperature and freezing rate have been demonstrated on cellulose cryogels [98] cooled at −68 °C and −40 °C. Smaller pore sizes were obtained at the lower temperature (−68 °C) due to the higher cooling rate. Cryogels with the highest specific surface area of 201 m^2^/g (i.e., the smallest pore size) were obtained at −196 °C [98]. The opposite approach (a low cooling rate) is used to increase the lightness of the cryogel [98]. Optimum freezing conditions can be determined by the initial crystallization temperature of the solvent and the freezing point for each polymer solution [99].

The structure of the cryogel will also be influenced by the type of cellulose solvent and the inclusion of various fillers or additives. Cryogel scaffolds often have more than one component and can consist of mixtures of two or more polymers or composites. Composite cryogels can be produced using both polymers and additives (nanoparticles and fibers) to obtain a material with improved physical, chemical, and biological properties. These cryogels can combine the beneficial properties of each component [59]. For example, cryogels of carrageenan/cellulose nanofibrils as carriers of antimicrobial α-aminophosphonate derivatives were produced by crosslinking with glyoxal. Cellulose nanofibrils significantly strengthened the composite material, improving its mechanical properties. Scaffolds of this material have been proposed for use as antimicrobial wound-healing materials and have been shown to be effective against *Staphylococcus aureus* infection [100].

Composite nanocellulose/gelatin cryogels with controlled porosity and network structure and good biocompatibility were obtained by chemical crosslinking of dialdehyde starch and subsequently used as carriers for the controlled release of 5-fluorouracil [101]. An increase in nanocellulose content (from 0.5 to 5 parts relative to gelatin) increased the specific surface area and porosity of the composite cryogel. The swelling coefficients first increased and then decreased with an increase in the nanocellulose content. Increasing the nanocellulose content resulted in improved drug loading and crosslinking rates.

The next section provides information on a variety of cellulose cryogels and cellulose-based composite cryogels produced using different solvents. The quality characteristics of the produced cryogels and their applications for biomedical purposes are presented.

## 4. Cellulose-Based Cryogels and Their Applications in Biomedicine

Cellulose cryogels, as a new generation of porous materials, are of great interest in tissue engineering, as they offer new solutions and improve existing systems and procedures [3]. In addition to their high porosity and mechanical strength, cellulose cryogels can be modified to enhance the attachment of certain other materials (e.g., extracellular matrix proteins, cultured cells, or chemicals) that can promote cell immobilization and growth [102,103] on cryogel scaffolds. Table 2 provides data on cellulose-based cryogels obtained by freeze-drying using various solvents and includes the main characteristics of the cryogels and the possible directions of their biomedical applications (Table 2).

The use of various solvents and cellulose dissolution techniques has produced cryogels with suitable properties, morphology, and mechanics for biomedical applications. Further, cellulose-based cryogels have shown good sorption properties; for example, keratin/cellulose cryogels have been successfully fabricated for the adsorption of oil/solvent [115]. Highly porous (more than 90%) and ultra-light (density less than 0.035 g/cm^3^) cellulose/biochar cryogels have also shown high sorption capacities. The addition of 5% biochar to a cellulose cryogel yielded the highest sorption capacity, at 73 g/g of petroleum [116]. Cryogels formed from hydroxypropyl methylcellulose (HPMC) and bacterial cellulose nanocrystals (CNC) have shown good adsorption of organic pollutants [117]. Shapable cellulose nanofiber/alginate cryogels with underwater super-elasticity have been used for protein purification [118]. Highly porous (94.7–97.1%) light (density 0.016–0.028 g/cm^3^) hydrophobic cellulose cryogels (unbleached long fiber of *Pinus elliottii*) have shown a high homogeneous sorption capacity (65.18 g/g) and heterogeneous sorption capacity (68.42 g/g) (solvent organosilane methyltrimethoxysilane) [119]. Thus, cellulose cryogels can be produced with different microstructures and properties, and varying the conditions of cellulose dissolution and the parameters for producing cryogels can result in cryogels with many different desirable qualities.

## 5. Conclusions

Due to the advantages of the freeze-drying method, interest is growing in the production of polysaccharide-based porous materials by cryogelation. The use of natural polymers for the production of cryogels, in contrast to synthetic polymers, makes it possible to create biocompatible medical materials (scaffolds) with a minimal immune response. Cellulose, due to its availability, renewability, non-toxicity, and biocompatibility, is a promising raw material for producing cryogels for biomedical applications. The production of cellulose cryogels by freeze-drying is a promising and steadily developing direction in tissue engineering. Cellulose cryogels have unique properties imparted by their interconnected super-macroporous structure and mechanical stability that make them attractive materials for a variety of applications. Much research has focused on the development of cellulose cryogels for tissue engineering. The results show that cellulose cryogels are promising tools and are applicable as scaffolds for various tissue types.

Physical and chemical parameters affect the formation of cryogels, such as the origin of the cellulose, dissolution parameters, type of solvent, temperature, freezing rate, and the inclusion of various fillers. Varying the parameters of cellulose dissolution, production technology, and freezing can change the properties of the cryogels and set the desired final characteristics of the product. Due to its complex supramolecular structure, cellulose is difficult to dissolve. Thus, an important task remains the selection of a cellulose solvent that can be easily removed from the final product prior to its use for biomedical purposes. The production of composite cryogels is promising for imparting additional properties to the cryogel (changes in morphology and mechanical properties). An important direction for research in the field of cryogels is the preservation of the properties of cryogels during their use. Cellulose cryogels have huge potential in the repair and regeneration of various tissue types, including cartilage tissue, bone tissue, and nerves, in wound healing, and in the delivery of controlled release drugs.

## Figures and Tables

**Table 1:** Characteristics of typical cellulose solvents.

Solvent	Advantages	Disadvantages	Reference
LiCl/DMAc	It does not cause any destruction of the cellulose, provided that destructive pretreatments are avoided (such as heating over 80 °C).	The difficulty of removing LiCl from the final products.	[45]
Ionic liquids	They completely dissolve the material’s components.	Ionic liquids do not evaporate, have low volatility, which complicates their regeneration.	[50,51,52,53]
7–9%NaOH/water (7%NaOH/12%urea/water)	Cellulose gels can be obtained.	The thermodynamic quality of the solvent decreases with increasing temperature, as the number of cellulose–cellulose interactions increases more rapidly than the number of cellulose–solvent interactions; Na+ ions penetrate deeply into the cellulose structure, making it difficult to remove alkali.	[26,42,43]
Complexing compounds of Cu with ethylenediamine (or Cd-ethylenediamine complexes)	Commonly used to determine the molecular weight of cellulose.	The difficulty of removing from the final products.	[44]
*N*-methyl-morpholine-*N*-oxide monohydrate	Direct solvent of cellulose: *N*-methylmorpholine-*N*-oxide (NMMO) is a cellulose solvent used industrially for the spinning of cellulosic fibers (the Lyocell process). NMMO is known to change the highly crystalline structure of cellulose after dissolution and regeneration.	In theory, this dissolution process is merely physical, but in practice many side reactions might occur.	[46,47,48,49]
Concentrated phosphoric acid	Rapid dissolution, easily removed and regenerated.	Causes significant destruction of macromolecules.	[54]

**Table 2:** Cellulose cryogels for biomedical applications.

Polymer	Production	Characteristics	Application	Reference
MCC	Calcium thiocyanate tetrahydrate and water (117 °C)	Porosity 94.3% Density 84.1 kg/m^3^ Surface area 23 m^2^/g E 13.27 ± 1.5 МРа	New filter types, various biomedical applications.	[31]
MCC	8 wt% NaOH-water (cross-linking with epichlorohydrin)	Pore size up to 200 µm Density 0.04–0.121 g/cm^3^	Drug release, materials with controlled morphology and porosity.	[33]
MCC/pectin	1-Allyl-3-methylimidazolium chloride	Dense network structure	Hemostatic material (had no effect on cell proliferation but offered favorable properties in liver hemostasis).	[104]
HEC	Cryogenic treatment with citric acid, freeze-drying	Interconnected pores 100–180 µm	Matrices for immobilized enzymes and cells, readily degraded in acidic conditions	[105]
HEC/polyaniline	Stirred at 40 °C in water for 20 min, sonicated		tissue engineering scaffolds, high survival and proliferation in electric field, good adhesion, spreading, and rearrangement onto materials.	[106]
CMC	Dissolved in deionized water and crosslinking with adipic acid dihydrazide and a small excess of the carbodiimide at −20 °C.	E 4.2 ± 1.4 MPa	Neural tissue engineering, cell delivery (restoration of brain tissue through delivery to the neural network).	[16]
CMC/Col	Mixing two streams: CMC solution (2%) in deionized water with adipic acid dihydrazide, buffer solution and solution N-(3-dimethylaminopopyl)-N′-ethylcarbodiimide chloridate (EDC, in deionized water). The resulting cryogels were soaked in the collagen solution, and then soaked in the EDC solution to fix the collagen.	Porosity > 90% Uniform density	Tissue engineering, spreading and proliferation of NOR-10 fibroblasts.	[107]
CMC/Col CMC/Col/TCP	Mixing two solutions (1:2)-CMC solution (distilled water), Col solution (acetic acid). TCP was added to the final solution.	Average lamellar spaces 204 ± 95 µm (Col/CMC) and 195 ± 21 µm (Col/CMC/TCP) E 309 ± 18 kPa (Col/CMC) and 481 ± 27 kPa (Col/CMC/TCP)	Regeneration of hard tissues, non-toxic and compatible with blood.	[108]
CMC/PVA/honey	Solvent water, each layer was applied alternately with preliminary freezing of the previous.		Wound healing, showed activity against *S. aureus* compared to their counterparts without honey.	[109]
CNF (bleached softwood kraft pulp)	Mechanical defibrillation in deionized water, sonication to obtain the nanofibril aqueous gel, which then sprayed and atomized at 40 MPa, frozen in liquid nitrogen and freeze-dried.	Density 0.0018 g/cm^3^ Surface area 389 m^2^/g	Tissue engineering, evaluated using 3T3 NIH cells.	[110]
CNF (bleached birch Kraft Pulp)	Solvent-TEMPO, sodium bromide, NaOH. TEMPO-oxidized cellulose fibers (NaClO) were precipitated in ethanol. CNF hydrogels were obtained from the CNF films followed by solvent exchange from ethanol to tertbutanol, frozen in liquid nitrogen, and freeze-dried.	Porosity 88.0–99.7% Pore size 10–200 µm Density 0.004–0.180 g/cm^3^ Surface area 158–308 m^2^/g E 28–104.4 kPa	Tissue engineering, evaluated using HeLa and Jurkat cells.	[111]
CNF (cellulose powder)	CNF powder in deionized water dispersed by sonication, crosslinked with glyoxal solutions, frozen in liquid nitrogen, freeze-dried.	For CNF cryogel 35 ± 9 µm, for crosslinked cryogel 60 ± 20 µm 0.003–0.11 g/cm^3^ for CNF cryogel, 0.003–0.09 g/cm^3^ for crosslinked cryogel Up to 1 m^2^/g 0.1 MPa for CNF cryogel, 50.8 ± 8 MPa for crosslinked cryogel	Bone tissue engineering, assayed in vitro with MG-63 cells.	[15]
CNF/Col (wood powder of 60–80 meshes)	NCFs were sonicated, oxidized by NaIO_4_. The dialdehyde NCFs were mixed with collagen 1:1, frozen and freeze-dried.	Porosity 90–95% Density 0.02–0.03 g/cm^3^	Tissue engineering, supported fibroblast proliferation.	[18]
CNF/gelatin/chitosan (high-purity softwood cellulose)	Crosslinking in situ with genipin, frozen and freeze-dried.	Porosity 95% Pore size 75–200 µm Density 0.06–0.09 g/cm^3^ E 1–3 MPa	Cartilage tissue engineering (ASC and L929 cells)	[17]
CNF/ bioactive glass	Cellulose nanofibrils (CNF) are introduced.	High porosity Pore size 96–168 µm E 24 ± 1 kPa	Bone tissue engineering (MC3T3-E1 cells and calvarial bone defect in rats in vivo)	[112]
CNF/PVA (commercial CNF)	Crosslinking with polyamide-epichlorohydrin, frozen in liquid nitrogen, freeze-dried.	Porosity 88.5–95.3%Pore size 90 and 20 µm Density 0.006–0.05 g/cm^3^ Compressive strength 5–220 kPa E 0.04–8.3 kPa	Skin tissue engineering, supported fibroblast cells.	[113]
CNF)/ NIPAm (commercial bleached softwood kraft pulp)	Crosslinked and sonicated, frozen in liquid nitrogen, freeze-dried.	Density 0.01–0.14 g/m^3^	Drug release.	[114]
Cellulose (wood dust from the plywood sanding)	Nanocellulose suspension from alkaline treated wood waste powders was redispersed in deionized water, frozen and freeze-dried.	Porosity 97.8–99.8% Pore diameter 3.7–8.3 nm Density 0.004–0.036 g/m^3^ Surface area 419–457 m^2^/g, E 7–165 kPa, Thermal performance 34–44 mW/m⋅K	Biomedicine, pollution filtering, thermal insulation.	[77]

MCC—microcrystalline cellulose, ECH—epichlorohydrin, HEC—hydroxyethylcellulose, CMC—carboxymethyl cellulose, ECM—extracellular matrix, EDC—*N*-(3-dimethylaminopopyl)-*N*′-ethylcarbodiimide chloridate, Col—collagen, TCP—tricalcium phosphate, TEMPO—2,2,6,6-tetramethylpiperidin-1-yl oxyl, PVA—polyvinyl alcohol, CNF—cellulose nanofibril, NIPAm—*N*-isopropylacrylamide.

## Data Availability

Not applicable.
